# Triple cancer of gallbladder, common bile duct and papilla of Vater: Report of a case and review of literature

**DOI:** 10.1016/j.ijscr.2021.106469

**Published:** 2021-10-06

**Authors:** Stefano Passoni, Takamune Yamaguchi, Emilie Uldry, Emmanuel Melloul, Nermin Halkic, Alessandra Cristaudi

**Affiliations:** a Department of General Surgery, Regional Hospital of Locarno, Via all'Ospedale 1, 6600 Locarno, Switzerland; b Hepato-Biliary-Pancreatic Surgery Division, Department of Surgery, Graduate School of Medicine, The University of Tokyo, 7-3-1 Hongo, Bunkyo-ku, Tokyo, Japan; c Department of Visceral Surgery, University Hospital of Lausanne, Rue de Bugnon 46, 1011 Lausanne, Switzerland; d Department of Visceral Surgery, Regional Hospital of Lugano, Via Tesserete 46, 6903 Lugano, Switzerland

**Keywords:** Biliary tract cancer, Hepatic and pancreatic surgery, Gallbladder disease, Multiple cancer, Case report

## Abstract

**Introduction and importance:**

Synchronous malignancies of gallbladder and biliary tree are together rare entity whose pathogenesis is yet unknown. We report the case of a triple synchronous cancer of 3 distinct location: gallbladder, common bile duct (CBD) and papilla of Vater.

**Case presentation:**

An 84-years-old woman, was admitted to our Hospital with clinics features of obstructive jaundice.

Dilatation of the biliary tree and CBD without evidence of gallstones was seen at US. CT scan confirmed distal CBD obstruction.

An *endo*-US showed a nodule of the head of pancreas infiltrating the lower CBD.

Finally, hepatic-MRI displayed a gallbladder malignancy with invasion of CBD.

Preoperative staging showed 3 diagnostic suspicions: carcinoma of CBD on CT, pancreatic carcinoma on *endo*-US and malignancy of gallbladder on MRI.

A cephalic duodenopancreatectomy and radical gallbladder resection was performed.

Final pathology revealed 3 distinct location of moderately differentiated adenocarcinomas: Gallbladder, CBD and Vater's papilla.

Microscopic examination didn't detect any direct continuity between the 3 tumors. Metastases were identified in the pancreaticoduodenal, peri-hepatic and peri-gastric lymph nodes.

**Clinical discussion:**

Literature displayed 22 cases of synchronous malignancies of gallbladder and CBD and 1 case of triple cancer with associated Vater's papilla carcinoma.

In most of these cases, an association with an anomalous pancreatic-bile duct junction was reported.

Although the real incidence remain unknown, it was reported to occur in 5–10% of CBD cancers.

**Conclusion:**

Suspicion of such combination of cancer should be remembered, especially when preoperative investigations don't allow a precise localization of tumor in the biliary tree.

## Highlights

• Rare case report of biliary tract cancer
• Diagnosis and treatment of multiple biliary tumors
• Differential diagnosis of biliary tract tumors

## 1 Introduction

Synchronous primary malignancies of gallbladder and common bile duct are a rare occurrence. Recent studies, mainly from Japanese authors, have shown that these lesions are usually associated with an anomalous pancreatic-bile duct junction (APBDJ) [1], [2]. On the other hand, Kurosaki et al. [3] reported that the presence of APBDJ is not an absolute necessity for the development of synchronous malignancies of the biliary tree.

It is also important to distinguish between synchronous malignancies with local metastasis from a primary cancer in the billiard tree. Warren et al. [4] and Gertsch et al. [5] have identified certain criteria to differentiate these two different clinical settings.

We report the case of a triple synchronous cancer of the gallbladder, common bile duct and papilla of Vater, the diagnostic path and surgical management.

This case report has been reported in line with the SCARE Criteria [include citation]' at the end of the introductory section [6].

## 2 Presentation of case

An 84-years-old Caucasian woman, known for hypertension, chronic renal failure and antecedent of hysterectomy, was admitted to our Universitary Hospital with abdominal pain and jaundice developing since a few days. The patient is in good general condition, independent, lives alone at home and does not report significant weight loss in the last period. No family history reported for other hepatobiliary and pancreatic tumors. On admission, blood samples showed an important cholestasis with a serum total bilirubin level of 222 micmol/l and serum direct bilirubin level of 190 micmol/l. Abdominal ultrasonography revealed dilatation of the intrahepatic biliary tree and of common bile duct (CBD), without evidence of gallstones in the gallbladder.

**Fig. 1:**
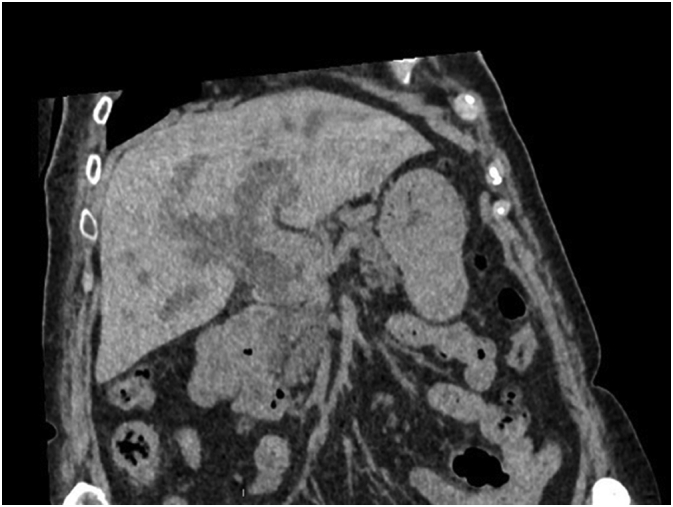
CT scan showing dilatation of intrahepatic biliary radicles with obstruction in the distal part of CBD.

Because of an acute renal failure due to hyperbilirubinemy, a non-contrast-enhanced CT scan was performed. This exam confirms an irregular dilatation of intrahepatic biliary tree with an obstruction in the distal part of common bile duct associated with pathologic local lymph nodes and a suspect lesion of segment IVb of the liver (Fig. 1).

**Fig. 2:**
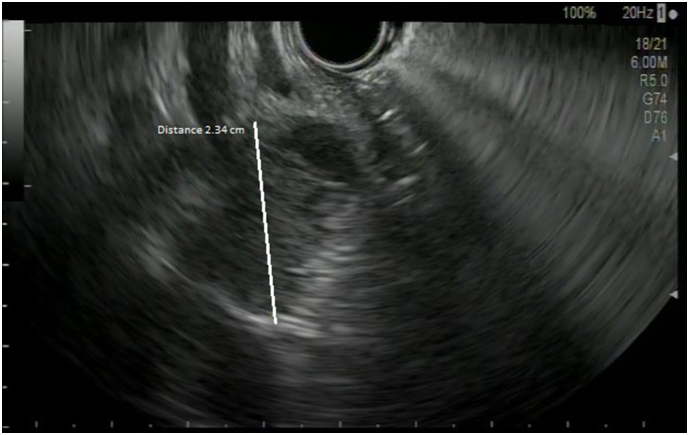
Endo-US showing a suspect nodule in the head of pancreas measuring 2.34 cm in diameter.

To avoid cholangitis, the patient rapidly underwent an endoscopic retrograde cholangiopancreatography (ERCP) with drainage of the lower CBD by a plastic stent. An endoscopic ultrasound (EUS) was performed at the same time, showing a nodule of the lower CBD infiltrating the head of pancreas and a biopsy of it was made (Fig. 2).

**Fig. 3:**
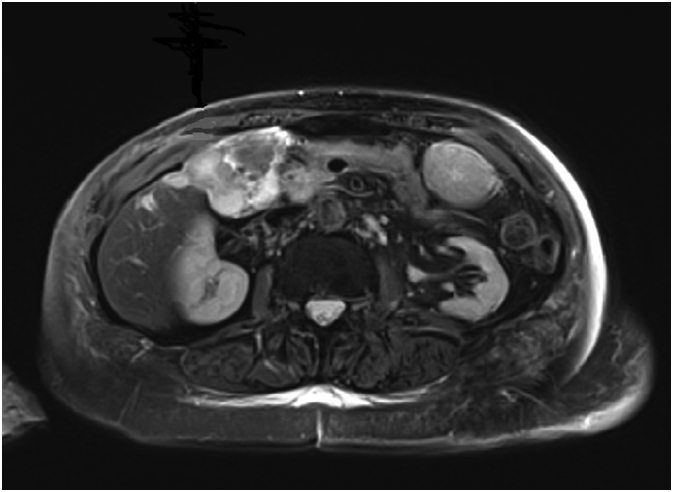
MRI with suspected gallbladder malignancy.

The latter showed a well-differentiated adenocarcinoma. We completed the pre-operative staging with a hepatic magnetic resonance that showed also a suspected malignancy of gallbladder with invasion of cystic duct and CBD (Fig. 3).

Tumor marker CEA was 4.5 μg/l and CA 19.9106 KU/l.

Facing three different diagnostic suspicions after radiological and endoscopic investigations (a pancreatic adenocarcinoma vs adenocarcinoma of the bile duct vs gallbladder adenocarcinoma), we decided to perform an explorative laparoscopy with wedge resection of IVb liver nodule showed in the MRI. No carcinosis neither malignancy were found at abdominal exploration and final pathology of the specimen respectively.

After multidisciplinary discussion and considering patient's will, the surgical approach was decided.

**Fig. 4:**
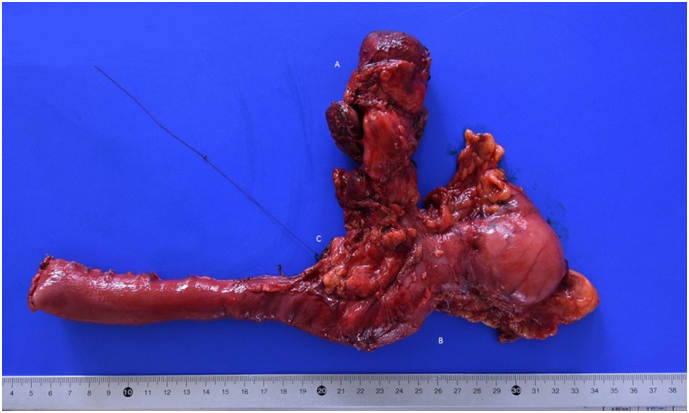
Surgical specimen with: A. Gallbladder B. Duodenum C. Head of pancreas.

The patient underwent a Whipple's cephalic duodenopancreatectomy, associated with a radical excision of gallbladder with hepatic resection of IVb and V segment, due to intraoperative suspicion of gallbladder cancer involving the CBD (Fig. 4). The intervention was performed by an expert surgeon specialized in hepatic, biliary and pancreatic surgery.

**Fig. 5:**
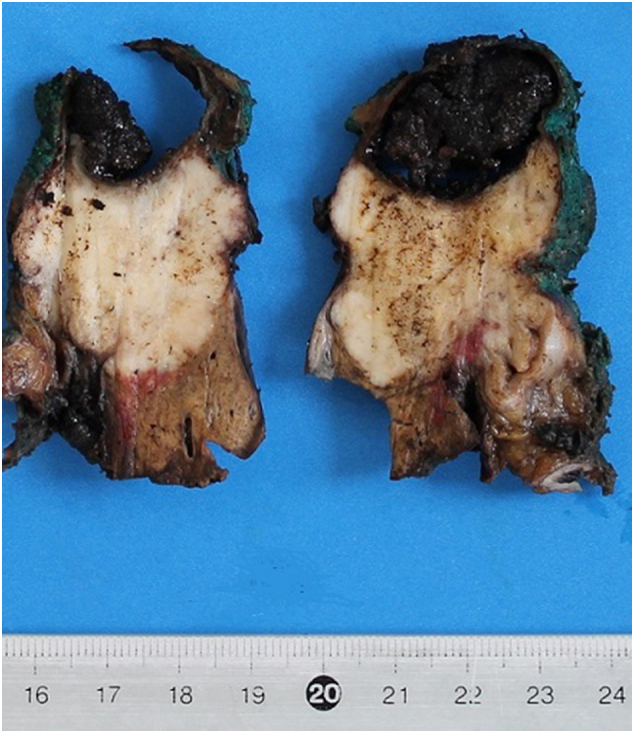
Adenocarcinoma of gallbladder.

**Fig. 6:**
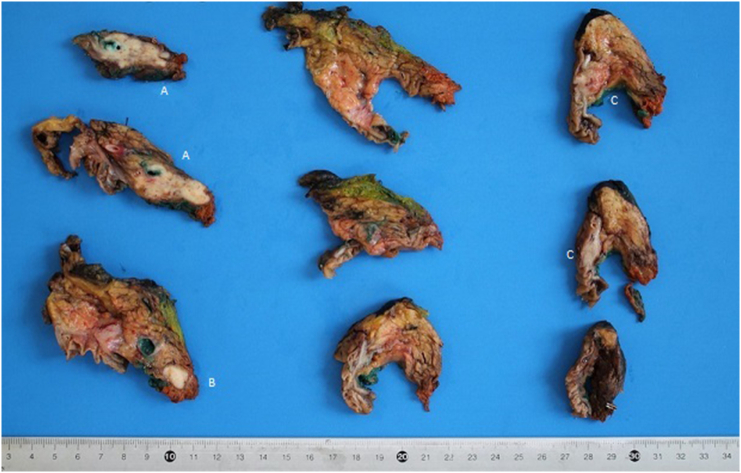
Surgical specimen with: A. Adenocarcinoma of CBD B. Metastatc lymph node C. Adenocarcinoma of papilla of Vater.

Final pathology report revealed the presence of three distinct cancers, respectively: moderated differentiated adenocarcinoma of gallbladder (Fig. 5) with invasion of cystic duct and hepatic tissue; moderately differentiated adenocarcinoma of mild CBD with serosa invasion (Fig. 6); Vater's papilla moderately differentiated adenocarcinoma with sub-mucosal extension (Fig. 6). Gallbladder and CBD adenocarcinoma showed perineural and microvascular infiltration. Microscopic examination didn't detect any direct continuity between the three tumors. Metastasis were identified in the pancreaticoduodenal, peri-hepatic and peri-gastric lymph nodes.

Immunohistochimical analysis showed defects in DNA mismatch repair (MMR) MLH1 and PMS-2 proteins, involved in the induction of p53 and apoptosis.

The pathological final staging was: pT3 pN1 L1 V1 Pn1 G2 R0 for the gallbladder adenocarcinoma; pT3 pN2 L1 V1 Pn1 G2 R0 for the CBD adenocarcinoma and pT2 pN2 L1 V0 Pn0 G2 R0 for the Vater's papilla adenocarcinoma.

The postoperative course was long, with a slow recovery by the patient. No significant complications were found.

After multidisciplinary discussion, in consideration of the general condition of the patient and the long postoperative recovery, it was decided for a clinical and instrumental follow-up.

## 3 Discussion

Synchronous malignancies of the gallbladder and biliary tree are rare. However, there are increasing reports, mainly from Asiatic authors, suggesting that this condition is not as rare as early reported, with an incidence of approximately 5–7.4% [3]. An explication for this, could be an inadequate sampling of the gallbladder specimen after resection of extra hepatic bile duct malignancies.

The pathogenesis of synchronous malignancies of gallbladder and extra-hepatic bile duct is yet unknown [7]. An important etiology is identified in an anomalous pancreatic-bile duct junction. This anomaly causes a perpetual reflux of pancreatic juice into the biliary tree that induces chronic inflammation and a higher cellular proliferation rate of the biliary ephytelium, increasing the risk of developing cellular mutations activating the carcinogenetic process [8]. In our patient the pancreatic-bile duct junction was difficult to be evaluated for the presence of the periampullary adenocarcinoma.

It is also important to distinct synchronous primary malignancies to metastasis from a primary elsewhere in the biliary tree. Warren et al. [4] and Gertsch et al. [5] have suggested 3 criteria to differentiate these two entities: 1) lack of anatomic continuity between the two tumors; 2) a growth pattern typical of a primary tumor; 3) clear histological differences between the two tumors. However in literature, these 3 criteria may not be sufficient to distingue synchronous tumors from metastasis. Kurosaki et al. [3] advised the mapping technique that consisted in detailed histopathologic analyses to clarify the mode of tumor spread, considering microvessel and perineural invasion, depth of tumor invasion and lymph node involvement. In our patient we find three distinct adenocarcinomas with a growth pattern typical of a primary tumor, with a perineural and microvessel invasion in the gallbladder and CBD adenocarcinomas, without any direct continuity between the two tumors; the adenocarcinoma of the papilla of Vater didn't have any microvascular or perineural invasion.

The distinction between metastasis and synchronous primary is an important prognostic factor in simultaneous lesions. In fact a better prognosis has been reported for synchronous primary lesions, if a complete R0 surgical resection is possible [3]. On the other hand, metastatic disease, perineural invasion, involvement of cystic duct and lymph nodes infiltration is obviously associated with a poorer prognosis. In both diseases, however, the average survival is low. In patients with synchronous primary it is about 50% at 3 years and 45% at 5 years while for metastatic patients it drops to 15% at 3 years and 10% at 5 years [9], [10].

## 4 Conclusion

Synchronous malignancies of gallbladder and biliary tree are rare conditions, but according to a more attentive surgical and pathological examination are now more common. Therefore it is important to keep a high index of suspicion, mostly when preoperative investigation don't allow a precise localization of the tumor in the biliary tree. When synchronous lesions are identified, looking for an anomalous pancreatic-bile duct junction as a possible cause and distinguishing primary lesions from metastasis, It's mandatory in order to correctly estimate the patient's prognosis.

Written informed consent was obtained from the patient for publication of this case report and accompanying images. A copy of the written consent is available for review by the Editor-in-Chief of this journal on request.

## Source of funding

None.

## Ethical approval

Patient consent.

## Guarantor

Stefano Passoni

## CRediT authorship contribution statement

Stefano Passoni writing the paper, Takamune Yamaguchy revision literature.

Emilie Uldry revision literature, Emmanuel Melloul revision literature, Nermin Halkic writing the paper, Alessandra Cristaudi writing the paper.

## Declaration of competing interest

None.
